# SUMOylation involved in malignant progression of multiple tumors and SENP5 may improve the chemotherapy sensitivity of hypoxic tumors

**DOI:** 10.3389/fphar.2025.1648271

**Published:** 2025-11-05

**Authors:** Chunyan Zhang, Bing Han, Yanxia Li, Yuxiang Wang, Min Liu, Zhongmin Jiang, Wenhan Wu, Xiaozhi Liu, Yafei Liu, Mingyong Liu

**Affiliations:** ^1^ Department of General Surgery, Tianjin Fifth Central Hospital, Tianjin, China; ^2^ School of Medicine, Tianjin University, Tianjin, China; ^3^ Tianjin Key Laboratory of Epigenetics for Organ Development of Premature Infants, Tianjin Fifth Central Hospital, Tianjin, China; ^4^ Tianjin Binhai Huangnan Plateau Medical Research Institute, Huangnan Tibetan Autonomous Prefecture People’s Hospital, Huangnan Prefecture, Qinghai, China; ^5^ Department of Pathology, Tianjin Fifth Central Hospital, Tianjin, China; ^6^ Department of General Surgery, Peking University First Hospital, Beijing, China; ^7^ Department of Anesthesiology, Peking University First Hospital, Beijing, China; ^8^ Department of Urology, Tianjin Fifth Central Hospital, Tianjin, China; ^9^ Institute of Emergency Medicine, Tianjin Fifth Central Hospital, Tianjin, China

**Keywords:** SUMOylation, tumor progression, hypoxic, SENP5, chemosensitivity

## Abstract

**Introduction:**

Small ubiquitin-related modifier (SUMO), a ubiquitin-like modification protein, is implicated in the aggressive progression of various tumor types. However, a comprehensive understanding of its mechanisms and the identification of therapeutically viable targets remain challenging.

**Methods:**

We analyzed the expression and clinical relevance of SUMO pathway components using public cancer databases (e.g., TCGA). The functional role of SUMOylation was investigated under varying oxygen conditions in vitro. The core SUMOylation enzyme UBC9 was inhibited pharmacologically with Spectinomycin B1 UBC9. The tumor-specific role of SENP5 was validated through genetic knockdown and overexpression in a panel of tumor cell lines and tumor-derived organoids. To assess the potential for off-tumor toxicity, the effects of UBC9 inhibition and SENP5 targeting were further evaluated in organoids derived from fetal mouse liver and kidney.

**Results:**

SUMO signaling was significantly activated in multiple tumors and correlated with poor prognosis. We demonstrated that oxygen levels modulate chemotherapy sensitivity via regulation of SUMOylation. While UBC9 inhibition broadly suppressed tumor progression through global deSUMOylation, it also induced toxicity in normal organoids. In contrast, SENP5 was specifically overexpressed in tumors and mediated selective removal of SUMO2/3 modifications, affecting a narrower range of substrates.

**Discussion:**

Our findings indicate that inhibition of the SUMOylation pathway is a promising therapeutic strategy. The broader implication of our study is that the precision and safety of this approach are contingent upon targeting specific components such as SENP5, which offers a superior therapeutic window by avoiding the adverse effects associated with global SUMOylation inhibition.

## 1 Introduction

Small ubiquitin-like modifier (SUMO) is a ubiquitin-like protein that can form covalent conjugates with specific lysine residues on target proteins, a process termed SUMOylation (Chang and Yeh, 2020). This reversible post-translational modification plays a crucial role in numerous cellular biological processes, including cell cycle progression, metabolism, gene transcription, DNA damage repair by modulating the stability, localization, and activity of substrate proteins. SUMOylation targets a wide range of proteins, such as transcription factors, signal transducers, viral proteins, and epigenetic regulators. The canonical SUMOylation consensus motif is ψKxE (where ψ is a hydrophobic residue, K is the target lysine, x is any amino acid, and E is glutamate) (Wilkinson et al., 2010). However, a significant number of substrates are modified on non-consensus lysines, facilitated by SUMO E3 ligases and non-covalent binding surfaces that enhance substrate recognition and conjugation efficiency beyond the primary motif (Knipscheer et al., 2007).In humans, three major SUMO paralogues exist: SUMO1, SUMO2, and SUMO3. SUMO maturation begins with Sentrin/SUMO-specific proteases (SENPs) cleaving the C-terminus to expos a diglycine motif. ATP-dependent activation leads to adenylation and transfer to the heterodimeric E1 activating enzyme (SAE1/SAE2), forming a high-energy thioester bond (Verde et al., 2025). SUMO is then conjugated to the E2 enzyme UBC9 (Chang and Yeh, 2020), which often recognizes the ψKxE sequence (Zhao et al., 2021), with further specificity provided by E3 ligases. DeSUMOylation is catalyzed by SENPs, which also recycle SUMO from modified substrates. In vertebrates, SENPs exhibit distinct substrate preferences: SENP1 and SENP2 process all SUMO paralogues; SENP3 and SENP5 prefer SUMO2/3; and SENP6 and SENP7 preferentially edit SUMO2/3 poly-chains (Youssef et al., 2023; Mendoza et al., 2003).

Dysregulation of SUMOylation is implicated in the pathogenesis of numerous diseases, including neurodegenerative disorders such as Alzheimer’s and Parkinson’s diseases, cardiovascular conditions, and immune dysfunctions (Yang et al., 2017). In cancer, SUMOylation influences tumor initiation, proliferation, metastasis, and therapy resistance by modulating central signaling pathways such as Wnt/β-catenin, NF-κB, and p53 (Lin et al., 2017). Furthermore, numerous receptors or intracellular signaling molecules can be modified by SUMOylation, such as insulin-like growth factor 1 (IGF-1R), type I TGF-β receptor (Lin et al., 2017; Liu et al., 2016) (in lung cancer), and Reptin and Potin (Liu et al., 2016; Wen et al., 2024; Diao et al., 2023) (in hepatocellular carcinoma and colorectal cancer), underscoring its role in tumorigenesis. Moreover, SENP-mediated deSUMOylation contributes to tumor development through alterations in transcriptional activity and signal transduction systems such as Wnt, NF-κB, and steroid hormone receptor systems.

Given these roles, the SUMO pathway has emerged as a promising target for drug development. Owing to the overexpression of *UBC9* in various malignancies, including melanoma, lung cancer, and ovarian cancer (Boix et al., 2022; Li et al., 2013; Lee-Law et al., 2021), making it a candidate for inhibition via its active site, E1 interface, or substrate-binding surface (Lee-Law et al., 2021). Arsenic trioxide, for instance, promotes SUMO1/2 chain formation on PML-RARα, inducing its degradation and differentiation of promyelocytic leukemia cells, and is now a standard therapy for acute promyelocytic leukemia (APL) (Yang et al., 2018; Cervia et al., 2023; Rabellino et al., 2012). Due to their substrate specificity, differential expression across tumors, and distinct activities toward SUMO paralogues, SENPs represent another attractive class of therapeutic targets.

Based on the updated public databases, this study applied bioinformatic tools to evaluate the activation status of the SUMO pathway across tumors, as well as its correlation with pathological subtypes, related signaling pathways, immune infiltration, and patient survival. Protein expression of key SUMO pathway members was further examined using tumor tissue microarrays. Our data demonstrate significant SUMO pathway activation in most cancer types, with elevated expression of certain members correlating with poor prognosis. Notably, SENP5 exhibited tumor-specific overexpression, and functional assays indicate that targeting SENP5 inhibits tumor organoid growth with fewer off-target effects compared to *UBC9* inhibition. These findings highlight SENP5 as a promising target for personalized cancer treatments.

## 2 Materials and methods

### 2.1 Data download preparation

Based on the UCSC Xena database at the University of California, Santa Cruz, RNA-Seq data and clinical-related data for 33 cancer types from the GDC TCGA cohort were downloaded from the Xena database (https://xenabrowser.net/datapages/), The datasets included: Gene expression RNAseq: “HTSeq FPKM (n = 151) GDC Hub”; Clinical traits: “Phenotype (n = 697) GDC Hub” and “Survival Data (n = 626) GDC Hub”. Additonal pan-cancer (PANCAN) data were also obtained, including: Phenotype: “Immune subtype”; Signatures: “Stemness score” (DNA methylation-based) from the Pan-Cancer Atlas Hub. Drug sensitivity data were obtained from the CellMiner™ database (https://discover.nci.mih.gov/cellminer/home.do).

### 2.2 Differential analysis of gene expression

The “HTSeq FPKM” RNA-data were processed and integrated using Perl. Expression levels of SUMO family genes across pan-cancer samples were visualized using boxplots. For cancers with at least five normal control samples, differential gene expression of SUMO family members was assessed using the Wilcoxon test. Significance levels were denoted as follows: ∗p < 0.05, ∗∗p < 0.01, ∗∗∗p < 0.001. A heatmap of significant results was generated using the R package “pheatmap”, and correlations among SUMO family genes were visualized using “corrplot”.

### 2.3 Signal pathway activity analysis

Reverse protein array (RPPA) data from the UCSC Xena database in TCGA was used to infer marker pathway activity scores for all tumor samples in TCGA, involving nine pathways related to cancer, including Apoptosis, Cell cycle, DNA damage response, epithelial mesenchymal transition (EMT), hormone androgen receptor (AR), hormone estrogen receptor (ER), Phosphatidylinositol-4,5-bisphosphate-3-kinase (PI3K)/protein kinase B (AKT), Rasopathes (RAS)/mitogen activated protein kinase (MAPK), and Receptor Tyrosine Kinase (RTK). The pathway score is the sum of the relative protein levels of all positive regulatory components minus the protein levels of the negative regulatory components in a specific pathway. Subsequently, the pathway activity score (PAS) was estimated (Dai et al., 2022). When PAS (high in the gene A group) > PAS (low in the gene B group), gene A is considered to have an activation effect on the pathway; otherwise, it has an inhibitory effect on the pathway (Horie et al., 2024).

### 2.4 Survival and prognostic analysis

Based on the Survival data (n = 626) GDC Hub data in the UCSC Xena database, we combined the mRNA expression data of SUMO family genes with the corresponding clinical survival data for 33 cancer types for expression survival analysis. The Kaplan-Meier method and log-rank test were used to evaluate the data for survival analysis (p < 0.05). Firstly, we divided the tumor samples into high expression and low expression groups based on the median value of gene expression levels, and then plotted survival curves using R packets of “survivor” and “survival”. Next, we applied a univariate COX risk proportional regression model to analyze the relationship between SUMO family gene expression and pan-cancer patient prognosis (Ye et al., 2018). Finally, the “survival” and “forestplot” in the R package were used to draw forest maps.

### 2.5 Immunological subtype analysis

Based on the “Immune subtype” data under the Phenotype item in the TCGA pan-cancer (PANCAN) of the UCSC Xena database, we used R packets “limma”, “ggplot2”, and “reshappe2” to perform immune subtype analysis on the SUMO family genes. We also used the Kruskal-Wallis (KS) test method (Tang et al., 2020) to detect the expression differences of SUMO family genes in different immune subtypes, with p < 0.05 being statistically significant.

### 2.6 Drug sensitivity analysis

Download drug sensitivity data based on the CellMiner™ database, and use R packages “input”, “limma”, “ggplot2”, and “ggpubr” to process and visualize drug sensitivity data. Correlation analysis was performed on the data using Cor. Test. p < 0.05 is considered to indicate significant drug sensitivity.

### 2.7 Cell lines and culture

CAKI-2 and HepG2 cell lines were obtained from Pricella Biotechnology. All cell lines underwent STR authentication and *mycoplasma* testing. Cells were cultured in manufacturer-recommended media (Pricella Biotechnology Co., Ltd., CM-0326, CM-0103), were maintained under standard conditions: 37 °C in a humidified atmosphere containing 5% CO_2_. All cultures were grown in 6-well or 12-well flat-bottom plates (Corning, catalog #3516 and #3513, respectively). For cryopreservation, cells were harvested, resuspended in cryopreservation medium, and gradually frozen before storage in liquid nitrogen. For revival, frozen cells were rapidly thawed, diluted in fresh medium, and cultured under standard conditions.

### 2.8 Cultivation and characterization of organoids

This study was approved by the Medical Ethics Committee of the Tianjin Fifth Central Hospital (No. WZX-EC-KY2022029), and written informed consent was obtained from all the patients prior to tumor sample collection. The collection of normal liver and kidney tissues from mice was approved by the Animal Ethics Committee of Tianjin Fifth Central Hospital (No. TJWZX2022012), in accordance with the Institutional Guidelines for Animal Welfare.

After excision, the specimens were immediately preserved in tissue storage solution and transferred to the laboratory under low temperature conditions. In a sterile environment, the tissue was minced into small fragments (3–5 mm^3^) and digested with collagenase. After digestion, the pellet was collected by centrifugation and mixed with matrigel. The mixture was then seeded into well plates and cultured in the appropriate medium. The medium was refreshed every 2 days, and the growth of the organoids was regularly monitored under a microscope. Once the organoids reached a diameter of 100–200 μm and approximately 80% of the culture area, they were collected for further passaging.

Then, the organoids were fixed in 4% paraformaldehyde for 1 h, pre-embedded in 3% agarose, and processed for paraffin embedding. Sections were prepared and subjected to hematoxylin and eosin (H&E) staining, immunohistochemistry, and immunofluorescence for detailed morphological and functional characterization.

### 2.9 Hypoxia and hyperoxia models

To simulate the hypoxic and hyperoxic tumor microenvironment, organoids and cell lines were cultured in a tri-gas incubator (Memmert, Germany) maintained at 5% O_2_ or 40% O_2_, 5% CO_2_, and balanced N_2_. Oxygen tension was continuously monitored and calibrated using the incubator’s integrated sensor. To ensure uniform oxygen penetration and avoid the formation of anoxic cores, organoid size was rigorously controlled to a diameter of <50 μm.

### 2.10 Protein extraction and Western blotting

Cells were lysed using RIPA Lysis Buffer (Strong) (Beyotime Biotechnology) containing PMSF. The lysis buffer was also supplemented with 1× protease inhibitor cocktail (Roche) and 20 μM PR-619 (a deSUMOylase inhibitor, Selleckchem) immediately before use. Lysates were clarified by centrifugation at 14,000 × g for 15 min at 4 °C, and the supernatants were collected for subsequent analysis. Proteins were separated by SDS-PAGE and transferred to polyvinylidene fluoride (PVDF) membranes. After blocking with BSA to reduce non-specific binding, the membrane was incubated with primary and secondary antibodies for specific detection. The following primary antibodies were used: anti-SENP5 (1:1,000, Abcam, ab58420), anti-Flag tag (1:2000, Cell Signaling Technology, 70,569), anti-UBC9 (1:1,000, Abcam, ab75854), and anti-β-Actin (1:5,000, Cell Signaling Technology, 4967S). HRP-conjugated secondary antibodies (anti-rabbit or anti-mouse, Cell Signaling Technology) were used at 1:2000. Finally, the enhanced chemiluminescence (ECL) was used to visualize target protein expression.

### 2.11 3D tumor organoid viability assay

Cell alarm™ Assay: Tumor organoids validated to retain the characteristics of their parent tissue were selected for this study. The organoids were digested using TrypLE, followed by centrifugation to collect the organoid pellets. A portion of the cell pellet was used for cell counting. The organoids were resuspended in 5% Matrigel and evenly seeded into a 96-well plate for standard culture. Once the organoids reached a stable logarithmic growth phase, they were treated with chemotherapeutic agents or exposed to varying oxygen concentrations. After the designated treatment period, cell viability was assessed using the Cell Titer-Glo 3D assay and the impact of different treatments on organoid viability was analyzed (Raghavan et al., 2021).

### 2.12 Lentiviral transduction assay

The lentiviral vector and Human SENP5-xFlag pHBLV-zsgreen + Puro virus (catalog no. HH20240614GX-LV01) were purchased from Hanheng Biotechnology (Shanghai, China). Vector refers to the empty xFlag-pHBLV-zsgreen + Puro vector containing a non-functional insert. First, tumor organoids were collected and digested with TrypLE to obtain single cells. The cells were subsequently infected with SENP5 and control lentiviruses at an MOI of 1. Following infection, cells were cultured in Matrigel supplemented with puromycin. Successfully infected organoids were expanded and used in the organoid formation assays.

### 2.13 Cell cycle analysis by flow cytometry

Cells and organoids stably overexpressing SENP5 were digested using TrypLE enzyme without EDTA, followed by centrifugation to collect cell pellets. The cells were washed with pre-cooled PBS and fixed in 70% ethanol for at least 4 h. After removing ethanol, the cells were stained with PI according to the manufacturer’s instructions. The stained cell suspension was analyzed using flow cytometry to determine the distribution of cells across different phases of the cell cycle.

### 2.14 TUNEL cell apoptosis detection assay

For adherent cell lines, after fixation, permeabilization, and endogenous peroxidase inactivation, the TUNEL reaction was performed to label DNA fragments, followed by detection with streptavidin-HRP and DAB substrates. The nuclei were stained with Hoechst, and TUNEL-positive apoptotic cells were visualized using a fluorescence microscope. For organoid spheres, a similar procedure was adopted with adaptations for proper handling to ensure thorough reagent penetration. After TUNEL labeling and detection, nuclei were stained with Hoechst, and images were captured under a fluorescence microscope to identify apoptotic cells within the organoids.

### 2.15 Statistical analysis

Data are presented as mean ± standard deviation (SD). To analyze the differences between individual groups, Student’s t-test or one-way analysis of variance (ANOVA) was performed using Prism GraphPad software. Statistical significance was set at p < 0.05.

## 3 Results

### 3.1 Pan-cancer analyses reveal molecular and clinical characteristics of SUMO pathway activity

**FIGURE 1:**
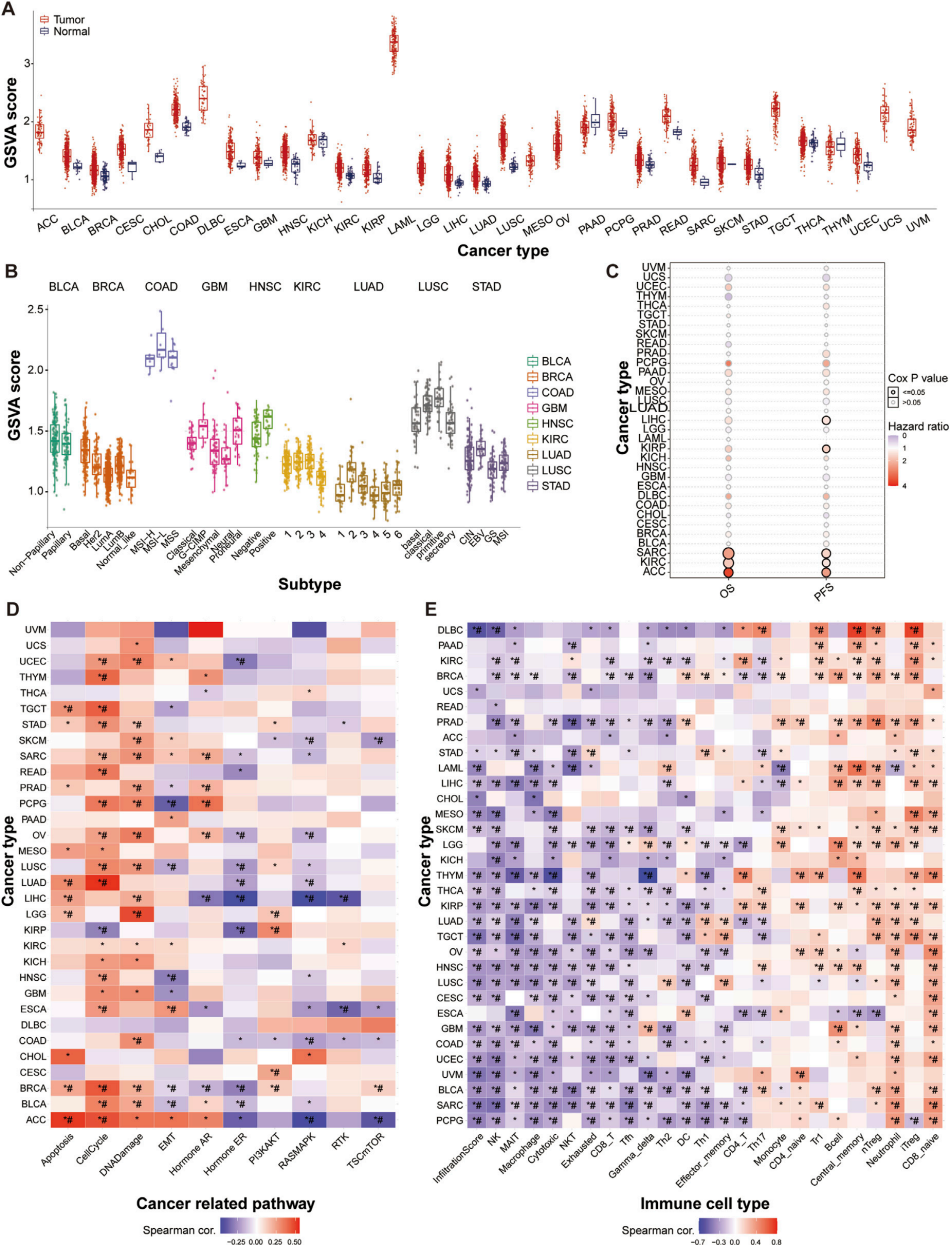
Widespread Activation of the SUMO Pathway in Tumors. **(A)** Pan-cancer hyperactivation of SUMO pathway as indicated by differential SUMO activity GSVA (Gene Set Variation Analysis) scores. ACC: Adrenocortical carcinoma; BLCA: Bladder Urothelial Carcinoma; BRCA: Breast invasive carcinoma; CESC: Cervical squamous cell carcinoma and endocervical adenocarcinoma; CHOL: Cholangiocarcinoma; COAD: Colon adenocarcinoma; DLBC: Lymphoid Neoplasm Diffuse Large B-cell Lymphoma; ESCA: Esophageal carcinoma; GBM: Glioblastoma multiforme; HNSC: Head and Neck squamous cell carcinoma; KICH: Kidney Chromophobe; KIRC: Kidney renal clear cell carcinoma; KIRP: Kidney renal papillary cell carcinoma; LAML: Acute Myeloid Leukemia; LGG: Brain Lower Grade Glioma; LIHC: Liver hepatocellular carcinoma; LUAD: Lung adenocarcinoma; LUSC: Lung squamous cell carcinoma; MESO: Mesothelioma; OV: Ovarian serous cystadenocarcinoma; PAAD: Pancreatic adenocarcinoma; PCPG: Pheochromocytoma and Paraganglioma; PRAD: Prostate adenocarcinoma; READ: Rectum adenocarcinoma; SARC: Sarcoma; SKCM: Skin Cutaneous Melanoma; STAD: Stomach adenocarcinoma; TGCT: Testicular Germ Cell Tumors; THCA: Thyroid carcinoma; THYM: Thymoma; UCEC: Uterine Corpus Endometrial Carcinoma; UCS: Uterine Carcinosarcoma; UVM: Uveal Melanoma. **(B)** Relationship between SUMO activity scores and tumor molecular subtypes. **(C)** Association of SUMO activity scores with patient survival outcomes. **(D)** Correlation of SUMO activation with pathway activity. **(E)** Correlation of SUMO activation with immune infiltration. Data are shown as mean ± SD; statistical significance is denoted as *p < 0.05.

We initially assessed the SUMO pathway activity across 33 different tumor types and observed pronounced activation of the SUMO pathway in nearly all tumors. Notably, GSVA (Gene Set Variation Analysis) scores of ACC, DLBC, LAML, LGG, MESO, OV, TGCT, UCS, and UVM normal tissues are almost zero, while their tumor tissue scores are at least twice higher than their corresponding normal tissues, indicating that SUMO pathway in these tumors is abnormally activated compared to normal tissues (Figure 1A; Supplementary Table 1). Analysis of SUMO pathway activity in relation to tumor subtypes revealed variability in SUMO pathway activation among different subtypes within same organ. Specifically, the G-CIMP and proneural subtypes of GBM, primitive subtype of LUSC, and EBV-positive subtype of STAD exhibited significantly higher SUMO activation than the other subtypes (Figure 1B; Supplementary Table 2). Correlation analysis between SUMO activation levels and patient survival revealed a significant poorer association between SUMO pathway activation and both overall survival (OS) and progression-free survival (PFS) in patients with ACC, KIRC, and SARC (Figure 1C; Supplementary Table 3). Subsequent analysis of the relationship between SUMO activation and gene signaling pathways demonstrated that SUMO activity is implicated in nearly all of the examined pathways, including apoptosis, cell cycle, DNA damage, EMT, and Hormone ER. Notably, SUMO activation had particularly pronounced effects on the cell cycle, DNA damage response, and apoptosis signaling pathways (Figure 1D; Supplementary Table 4). The immune cell infiltration landscape is an important parameter for assessing tumor malignancy and chemotherapy sensitivity (Wang et al., 2020; Xu et al., 2021). Our analysis showed that SUMO activation levels were negatively correlated with infiltration score, NK, MAIT, macrophage, cytotoxic, NKT, exhausted, and CD8-T cells, and positively correlated with CD8-naive, iTreg, neutrophil, nTreg, central memory, and B cells (Figure 1E; Supplementary Table 5).

### 3.2 The SUMO pathway members show a general trend of increased expression in various tumors

**FIGURE 2:**
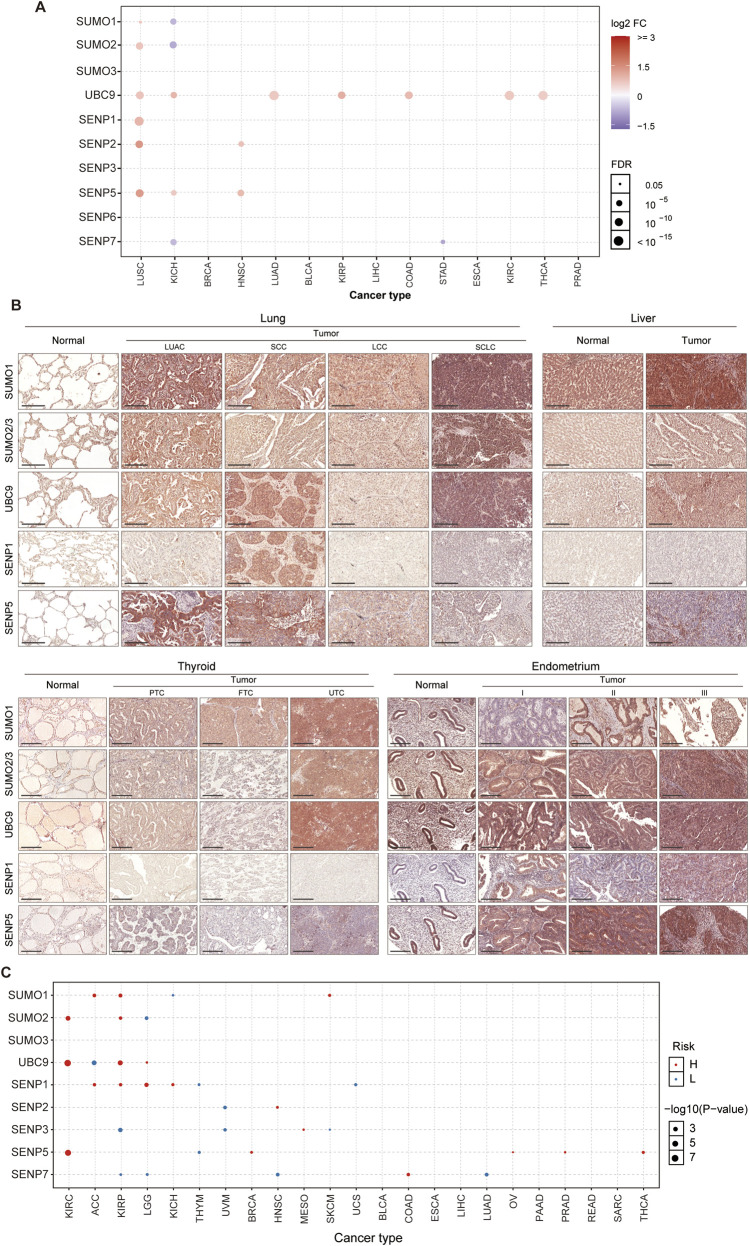
Prevalent Upregulation of SUMO Pathway Members in Tumors. **(A)** Increased mRNA expression of core SUMO pathway components in most tumor types compared to their matched normal tissues (except KICH). **(B)** Immunohistochemical validation (tissue microarray) demonstrating elevated protein levels of SUMO pathway members in four representative tumors versus corresponding normal tissues (Scale bar: 200 μm). LUAC: Adenocarcinoma; SCC: Squamous cell carcinoma; LCC: Large cell carcinoma; SCLC: Small cell lung cancer; PTC: Papillary thyroid carcinoma; FTC: Follicular thyroid carcinoma; UTC: Undifferentiated thyroid carcinoma. **(C)** Association between mRNA expression levels of SUMO pathway components and overall survival in cancer patients.

We subsequently examined the mRNA expression profiles of key SUMO pathway members across the 14 tumor types. Our analysis revealed that the mRNA levels of were significantly elevated in seven tumor types (LUSC, KICH, LUAD, KIRP, COAD, KIRC, and THCA) compared with their corresponding normal tissues. Among the six SENP genes evaluated, SENP5 was specifically upregulated in LUSC, KICH, and HNSC, SENP2 was specifically elevated in LUSC and HNSC, and SENP1 was notably overexpressed only in LUSC. In particular, LUSC exhibited markedly higher expression levels of SUMO1, SUMO2, UBC9, SENP1, SENP2, and SENP5 than normal tissues. HNSC also showed higher expression levels of SENP2 and SENP5 than normal tissues. Additionally, in KICH, UBC9 and SENP5 levels were elevated relative to control tissues, whereas SUMO1, SUMO2, and SENP7 levels were reduced compared to normal controls. Besides, UBC9 is the only SUMO pathway member significantly elevated in LUAD, KIRP, COAD, KIRC, and THCA tumors (Figure 2A).

Based on these bioinformatic analyses, we identified SUMO1, SUMO2/3, UBC9, SENP1, and SENP5 as key targets for further investigation. To validate these findings, we used tissue microarrays from patients with tumors and normal controls to assess the protein expression of these five SUMO pathway members in four common tumor types. Compared to normal lung tissue, lung adenocarcinoma (LUAC) exhibited highly elevated levels of SUMO1 and SENP5, whereas lung squamous cell carcinoma (SCC) showed increased expression of UBC9, SENP1 and SENP5. Small cell lung cancer (SCLC) demonstrated higher levels of SUMO1, SUMO2/3, and UBC9, whereas large cell lung carcinoma (LCC) showed markedly reduced SENP1 expression. In liver tissue, hepatocellular carcinoma exhibited elevated levels of SUMO1, SUMO2/3, UBC9, and SENP5 compared to normal controls. In thyroid tissue, papillary thyroid carcinoma (PTC) had increased SUMO1 and SENP5 expression, follicular thyroid carcinoma (FTC) showed higher SUMO1 levels, and undifferentiated thyroid carcinoma (UTC) exhibited elevated SUMO1, SUMO2/3, UBC9 and SENP5 levels. For endometrial cancers of different differentiation levels (stages I, II, and III), the expression of SUMO1, SUMO2/3, UBC9, SENP1, and SENP5 is significantly higher than that of normal tissues, except for SUMO1 in stage I and SENP1 in stage II, which show no significant changes compared to the normal group UBC9 (Figure 2B).

Additionally, we analyzed the association between the primary SUMO pathway members and overall patient survival. Our findings revealed that both elevated and reduced expression levels of UBC9, SENP1, SENP3, SENP5, and SENP7 may be associated with poor prognosis across various tumor types. Notably, high expression levels of SENP5 were specifically linked to adverse outcomes in patients with KIRC, BRCA, OV, PRAD, and THCA (Figure 2C; Supplementary Table 6). These results suggested that SENP5 may serve as a potential therapeutic target.

### 3.3 Oxygen levels significantly regulate protein SUMOylation and influence the chemotherapeutic sensitivity of tumor organoids

**FIGURE 3:**
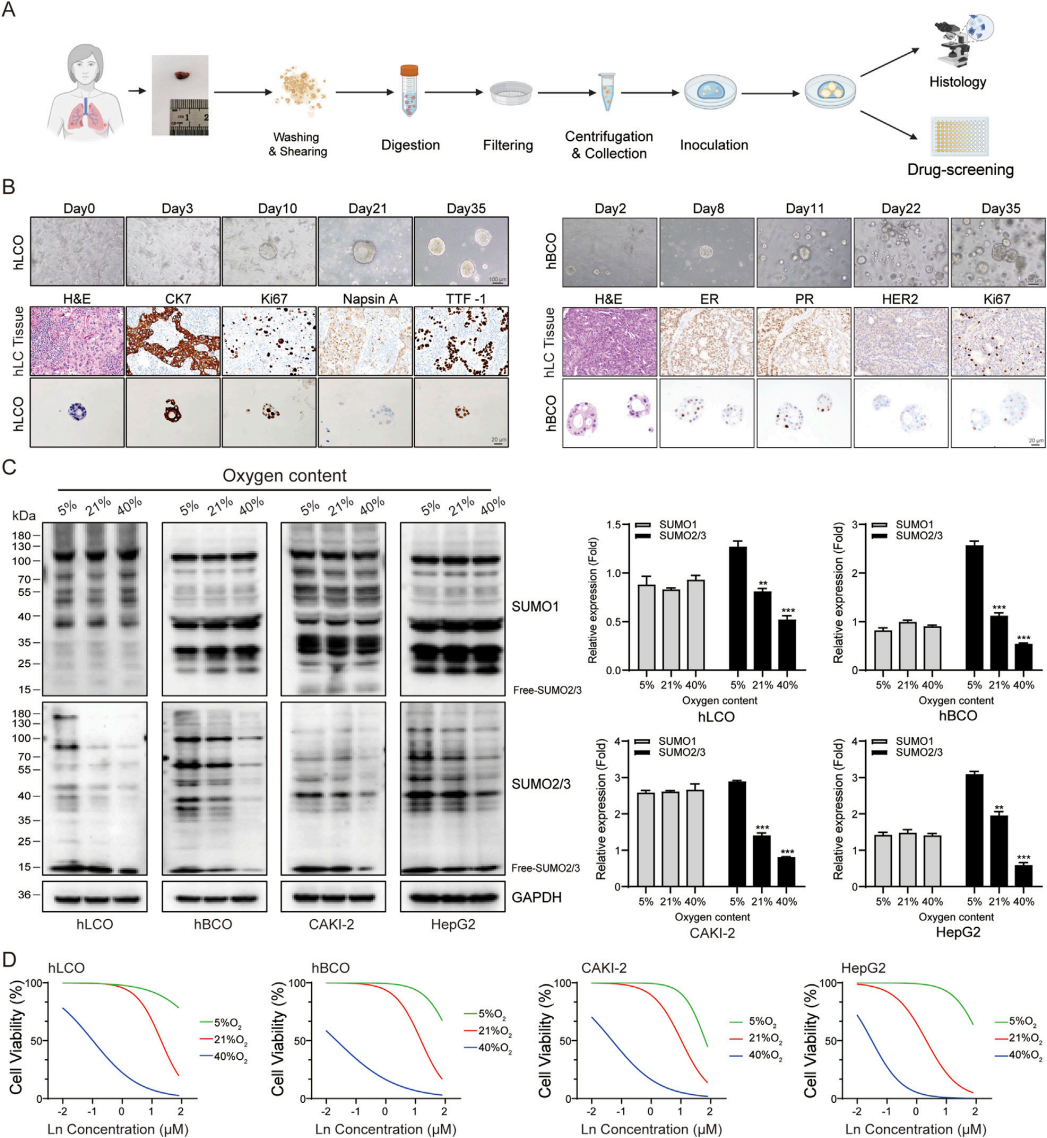
Oxygen Levels Modulate SUMOylation and Chemotherapy Sensitivity. **(A)** Schematic overview of tumor organoid culture system establishment and experimental workflow. **(B)** Morphological and molecular characterization of lung and breast cancer organoids. **(C)** Western blot analysis showing oxygen-dependent changes in SUMOylation levels in tumor organoids and cell lines. The free SUMO bands were excluded from the quantification analysis. **(D)** Cisplatin chemosensitivity assays in tumor organoids and cell lines under varying oxygen conditions. Ln on the x-axis represents the base-10 logarithm of the concentration (Log_10_ [Concentration, µM]). hLCO: Human lung cancer organoid; hBCO: Human breast cancer organoid; CAKI-2: Human renal clear cell carcinoma cell line; HepG2: Human hepatocellular carcinoma cell line. Data are presented as mean ± SD; statistical significance is denoted as *p < 0.05, **p < 0.01, and ***p < 0.001.

To assess the impact of protein SUMOylation levels on tumor chemosensitivity, we conducted a detailed analysis of breast cancer, lung cancer, renal clear cell carcinoma (KICH), and hepatocellular carcinoma models. First, we expanded existing lung and breast cancer organoids (established as outlined in Figure 3A) along with CAKI-2 and HepG2 cell lines. Morphological and molecular characterization of organoids confirmed their viability and phenotypic fidelity (Figure 3B). Subsequently, the two tumor organoids and two tumor cell lines were cultured under normoxic (21% O_2_), hypoxic (5% O_2_), and hyperoxic (40% O_2_) conditions, and subjected to Western blot analysis and chemosensitivity assays. Western blot analysis revealed that protein SUMOylation levels were significantly elevated in all four tumors under hypoxic conditions, whereas the opposite effect was observed under hyperoxic conditions (Figure 3C). Sensitivity to chemotherapy was notably reduced in hypoxic environments but significantly enhanced in hyperoxic environments across all four tumor types (Figure 3D).

### 3.4 Inhibiting UBC9 significantly suppresses tumor organoid growth but also leads to potential adverse effects

**FIGURE 4:**
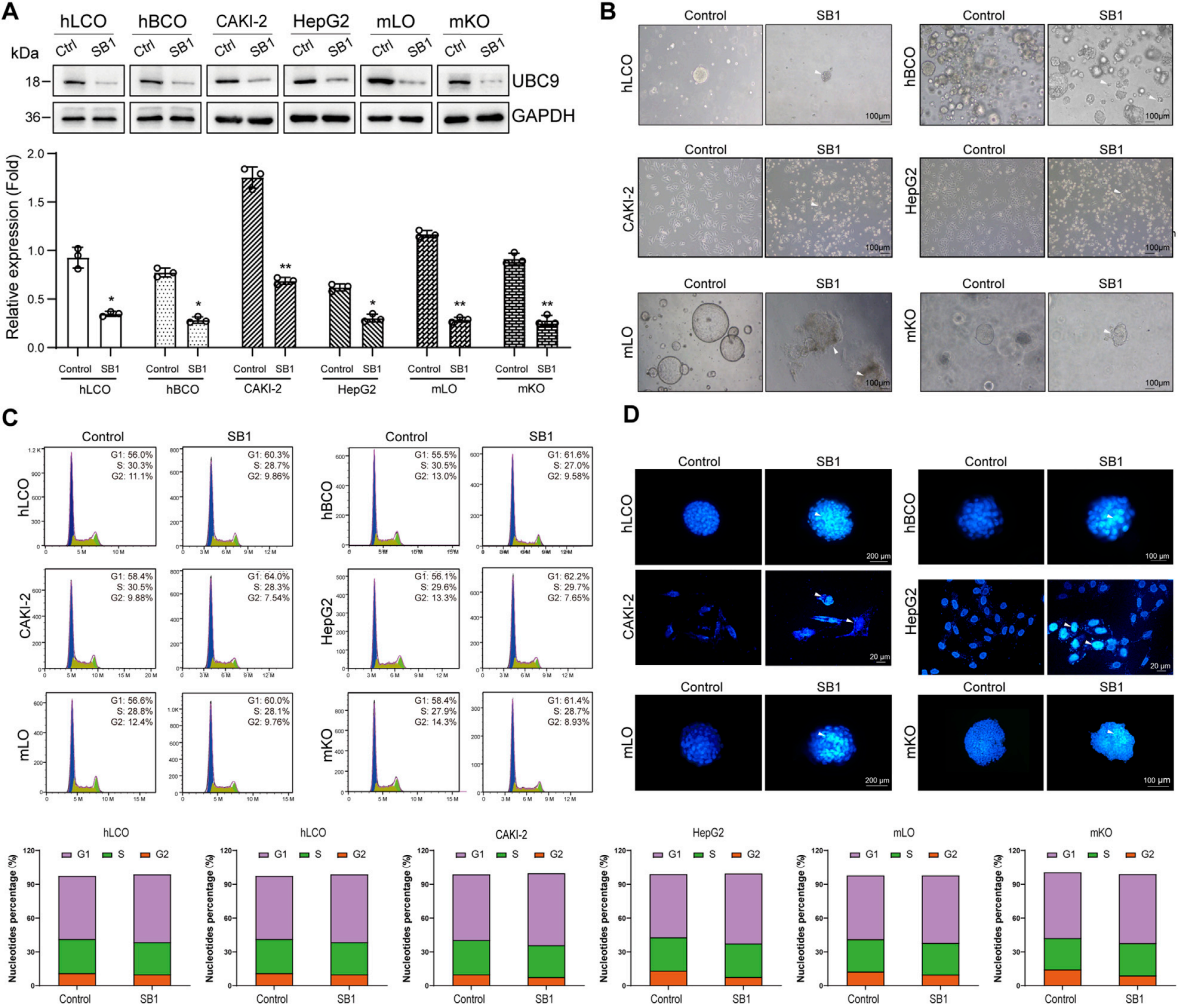
Global SUMO Interference Disrupts Tumor Malignancy and Induces Hepatorenal Toxicity. **(A)** Western blot analysis of UBC9 expression in four tumor organoid/cell line models and normal mouse embryonic liver/kidney organoids treated with the UBC9 inhibitor Spectinomycin B1 (SB1). **(B)** Bright-field images showing morphological changes (cell shrinkage and increased senescent cells) in SB1-treated organoids and cell lines. **(C)** Flow cytometry analysis of cell cycle arrest in SB1-treated organoids and cell lines. **(D)** TUNEL assay detecting apoptosis in SB1-treated organoids and cell lines. hLCO: Human lung cancer organoid; hBCO: Human breast cancer organoid; CAKI-2: Human renal clear cell carcinoma cell line; HepG2: Human hepatocellular carcinoma cell line; mLO: Mouse liver organoid; mKO: Mouse kidney organoid. Data are shown as mean ± SD; statistical significance is denoted as *p < 0.05, **p < 0.01, and ***p < 0.001.

In the present study, we inhibited UBC9 expression in tumor cells and organoids using the UBC9-specific inhibitor Spectinomycin B1 (SB1; Figure 4A). For comparative toxicity assessment, fetal mouse-derived liver and kidney organoids (culture and characterization results in Supplementary Figure S1) were also disturbed. Bright-field results showed that the growth of all cells and organoids was significantly inhibited, that is, the transparency of cells and organoids was dramatically reduced, and the number of adherent and senescent cells was increased (Figure 4B). Flow cytometry analysis showed that the cell cycle of both tumor cells and organoids, as well as that of mouse-derived organoids, was significantly arrested (Figure 4C). In addition, a significant increase in the proportion of apoptotic cells was observed in the TUNEL assay (Figure 4D). These results suggest that although inhibition of UBC9 can effectively suppress the growth of tumor-like organs, it also leads to significant liver and kidney damage.

### 3.5 Overexpression of SENP5 selectively promotes the growth of tumor organoids with minimal associated side effects

**FIGURE 5:**
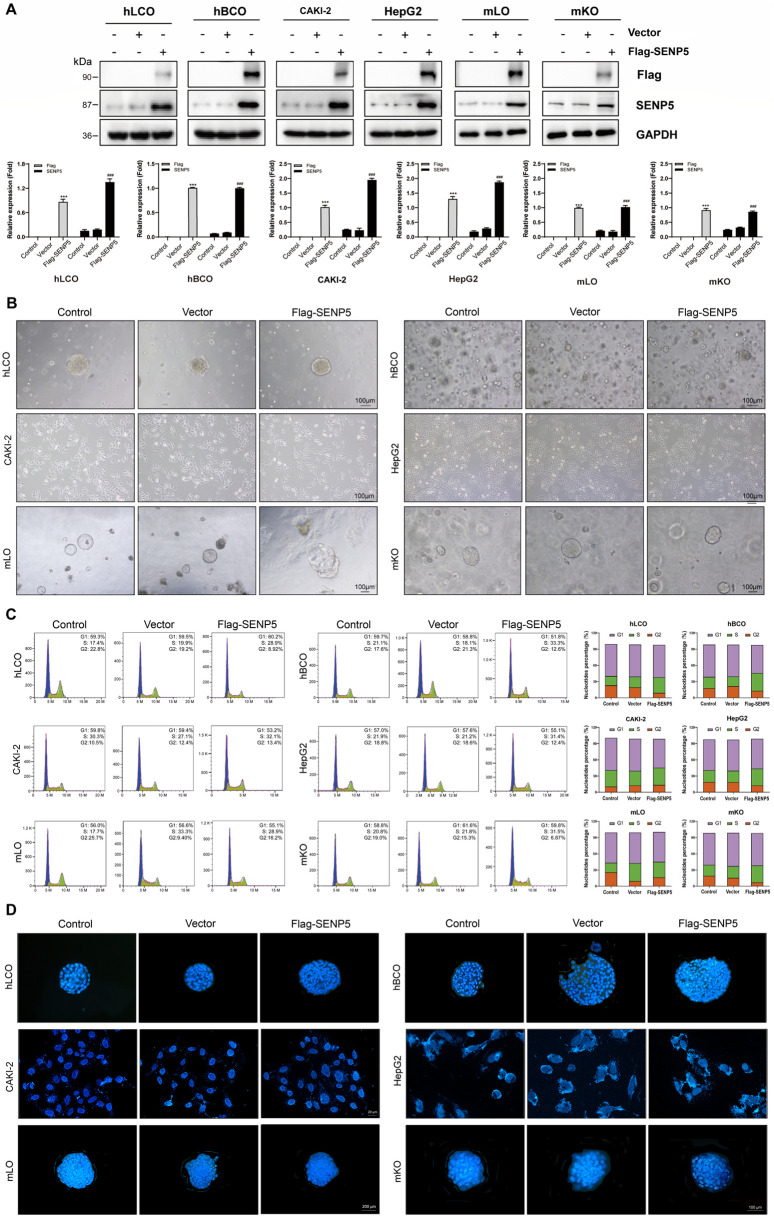
SENP5 Overexpression Suppresses Tumor Malignancy Without Apparent Toxicity. **(A)** Western blot analysis of SENP5 expression in tumor organoids/cell lines and normal mouse embryonic liver/kidney organoids following lentivirus-mediated SENP5 overexpression. **(B)** Bright-field images showing selective morphological alterations (cell shrinkage and senescence) in SENP5-overexpressing tumor organoids/cell lines, with no significant growth impact on normal embryonic liver/kidney organoids. **(C)** Flow cytometry analysis of cell cycle changes in SENP5-overexpressing organoids/cell lines. **(D)** TUNEL assay detecting apoptosis in SENP5-overexpressing organoids/cell lines. hLCO: Human lung cancer organoid; hBCO: Human breast cancer organoid; CAKI-2: Human renal clear cell carcinoma cell line; HepG2: Human hepatocellular carcinoma cell line; mLO: Mouse liver organoid; mKO: Mouse kidney organoid. Data are shown as mean ± SD; statistical significance denoted as *p < 0.05, **p < 0.01, and ***p < 0.001.

We first used lentiviral vectors to achieve overexpression of SENP5 in all organoids and cells (Figure 5A). Dynamic monitoring of spheroid diameters revealed that SENP5 overexpression promoted growth in CAKI-2 cells and breast cancer organoids but had minimal impact on lung adenocarcinoma organoids, HepG2 cells, and fetal mouse-derived liver and kidney organoids (Figure 5B, bright-field images). Flow cytometric cell cycle analysis further demonstrated that SENP5 overexpression significantly reduced the proportion of cells in G1 phase while increasing the population in G2/S phase across responsive models (hBCO and CAKI-2), indicating promotion of cell cycle progression and enhanced proliferation. In contrast, non-responsive models (hLCO, HepG2, and fetal organoids) showed no significant cell cycle distribution changes (Figure 5C). Additionally, TUNEL assays showed no significant increase in apoptosis across all organoids (Figure 5D).

### 3.6 SENP5 drives cell cycle pathway enrichment and enhances chemosensitivity of tumors under hypoxic conditions

**FIGURE 6:**
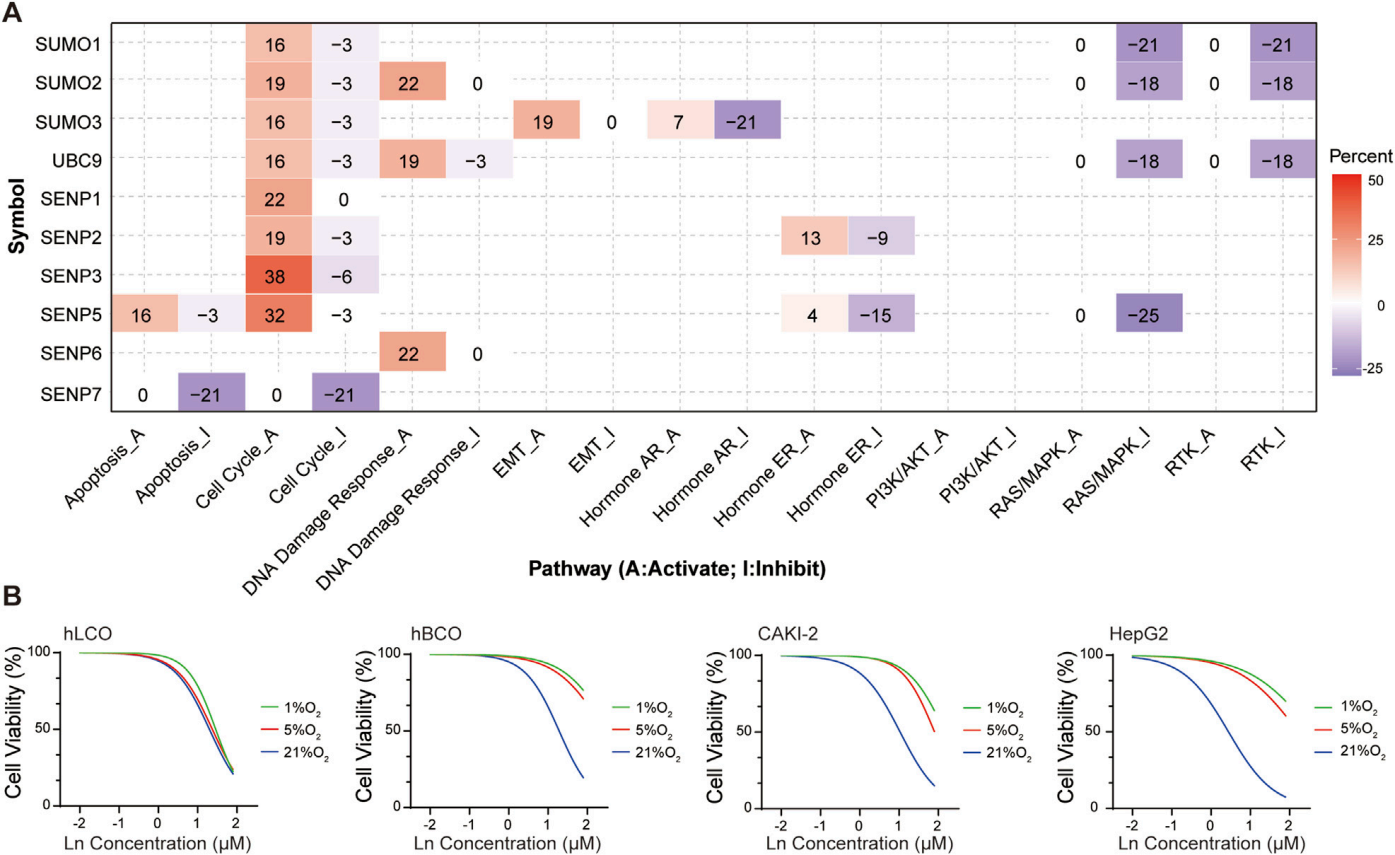
**(A)** Functional enrichment analysis of SUMO pathway components, highlighting SENP5-associated activation of the cell cycle pathway. **(B)** SENP5 overexpression potentiates cisplatin sensitivity in tumor organoids and cell lines. Ln on the x-axis represents the base-10 logarithm of the concentration (Log_10_ [Concentration, µM]). hLCO: Human lung cancer organoid; hBCO: Human breast cancer organoid; CAKI-2: Human renal clear cell carcinoma cell line; HepG2: Human hepatocellular carcinoma cell line. Data are shown as mean ± SD; statistical significance denoted as *p < 0.05, **p < 0.01, and ***p < 0.001.

Bioinformatics analysis revealed that key members of the SUMO pathway influence tumor progression by promoting the cell cycle, activating DNA damage responses, and inhibiting the RAS/MAPK and RTK pathways (Kubota et al., 2011). Specifically, SENP5 selectively activated the cell cycle pathway and inhibited the RAS/MAPK pathway (Figure 6A; Supplementary Table 7).

Although the tumor growth-promoting effects of SENP5 overexpression are undesirable, we hypothesized that its ability to enhance the cell cycle might be utilized to transition hypoxic tumor cells from a quiescent (G0) state to a proliferative phase (S and M phases), thereby increasing their susceptibility to chemotherapy. To test this hypothesis, we subjected tumor organoids and cells stably overexpressing SENP5 to hypoxic conditions and evaluated their sensitivity to cisplatin. The results demonstrated that SENP5-overexpressing tumor organoids and cells exhibited markedly increased chemotherapeutic sensitivity even under severe hypoxia (1% oxygen) (Figure 6B).

### 3.7 SENP5 modulates the RAS/MAPK pathway by regulating protein SUMOylation and phosphorylation

**FIGURE 7:**
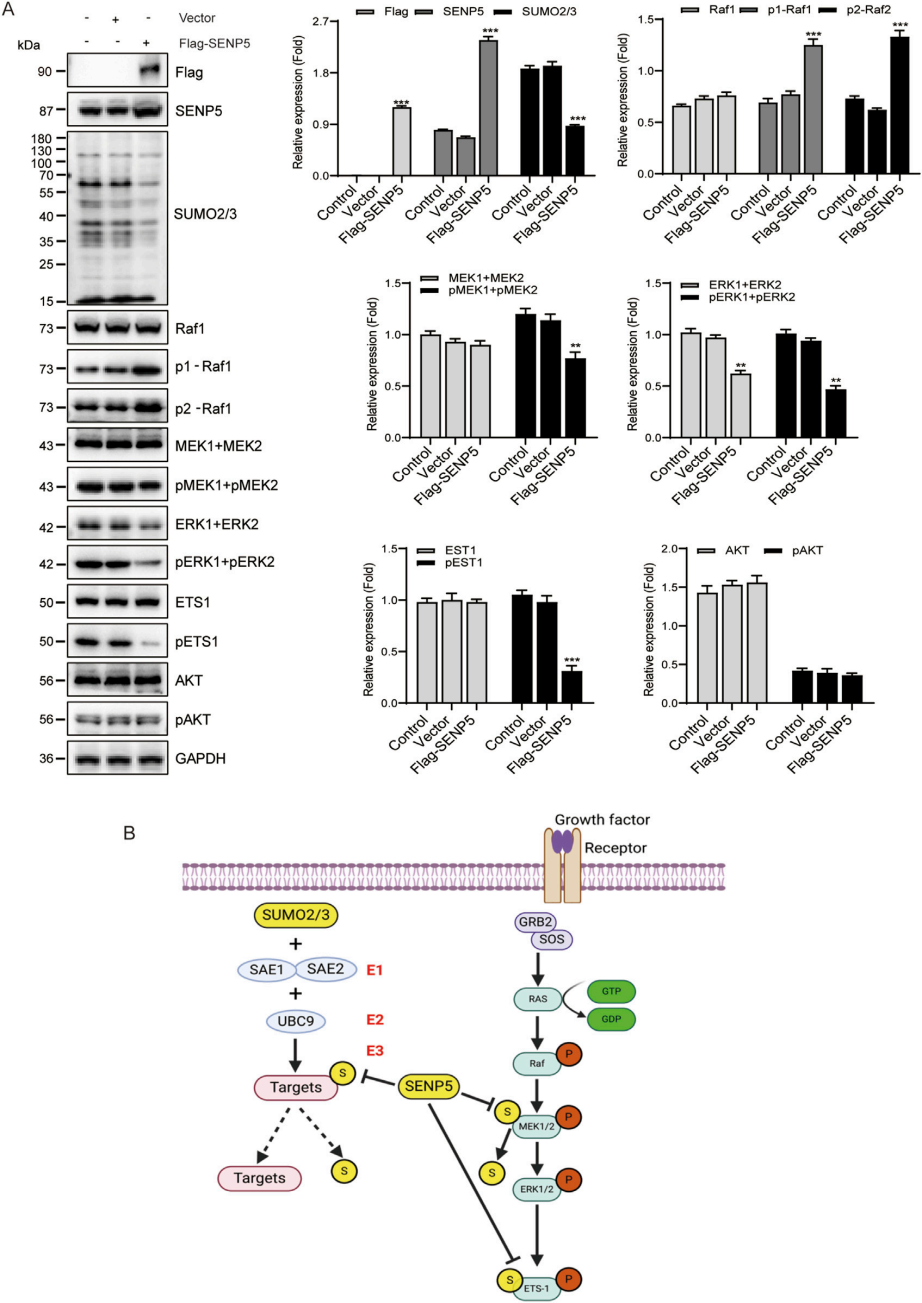
SENP5 Modulates the RAS/MAPK Pathway via SUMOylation and Phosphorylation Regulation. **(A)** Western blot analysis of key upstream and downstream molecules in the RAS/MAPK pathway following SENP5 overexpression. **(B)** Schematic diagram of the proposed molecular mechanism by which SENP5 regulates the RAS/MAPK pathway through SUMOylation and phosphorylation. Data are presented as mean ± SD (*p < 0.05, **p < 0.01, ***p < 0.001) where applicable.

Because SENP5 specifically inhibits the RAS/MAPK pathway, we investigated whether SENP5 affects this pathway by regulating SUMOylation and phosphorylation of target proteins. Our results demonstrated that SENP5 overexpression reduced the phosphorylation levels of MEK1/2, ERK1/2, and ETS1 while increasing the phosphorylation level of Raf1, with no significant effect on AKT phosphorylation in CAKI-2 cells (Figure 7A). These findings suggested that SENP5 may influence the phosphorylation of these proteins either through direct interactions or by modulating their SUMOylation levels (Figure 7B). However, the mechanism by which SENP5 enhances Raf1 phosphorylation was not elucidated in this study. Overall, these results indicate that SENP5 can specifically alter the RAS/MAPK pathway activity by regulating SUMOylation and phosphorylation of target proteins.

### 3.8 Screening of SENP5-Targeted drugs

**FIGURE 8:**
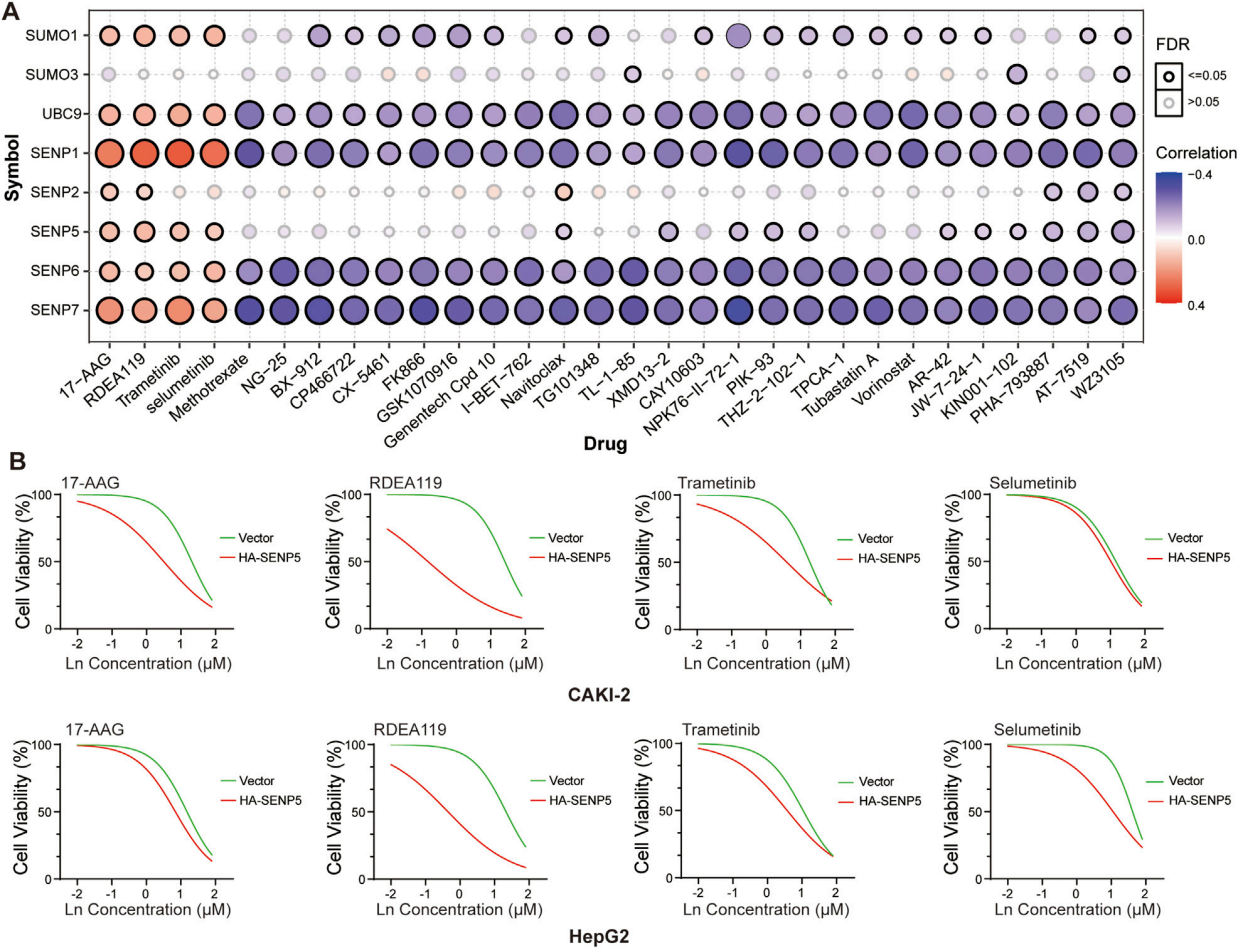
Drug Screening Targeting SENP5 Identifies Therapeutic Candidates. **(A)** Correlation analysis between drug sensitivity profiles (from the Genomics of Drug Sensitivity in Cancer, GDSC database) and SUMO pathway core components. **(B)** Sensitivity of renal and breast cancer models to selected drugs pre-versus post-SENP5 overexpression. Ln on the x-axis represents the base-10 logarithm of the concentration (Log_10_ [Concentration, µM]). Data are shown as mean ± SD (*p < 0.05, **p < 0.01, ***p < 0.001).

We next examined the correlation between drug sensitivity from the Genomics of Drug Sensitivity in Cancer (GDSC) database and the mRNA expression levels of key SUMO pathway members. Our analysis revealed that sensitivity to 17-AAG, RDEA119, Trametinib, and Selumetinib positively correlated with SENP5 mRNA levels (Figure 8A; Supplementary Table 8). For further investigation, we compared the drug sensitivity of SENP5-overexpressing CAKI-2 and HepG2 with that of control groups. The results showed that compared with the control group, CAKI-2 and HepG2 cells overexpressing SENP5 could significantly reduce cell viability under the action of the above four drugs, especially with a more significant effect on RDEA119. It can be seen that overexpression of SENP5 can significantly enhance the chemotherapy sensitivity of liver cancer and kidney cancer cells (Figure 8B).

## 4 Discussion

Members of the SUMOylation pathway, including SUMO family, E1 activating enzymes, E2 conjugating enzymes, and SENP de-SUMOylases, exhibit differential expression across various tumors. This variability complicates the comprehensive understanding of the mechanisms by which SUMOylation contributes to tumor progression, making it challenging to identify precise targets and develop suitable targeted therapies (Gu et al., 2023). Since SUMOylation is a crucial post-translational modification for thousands of mammalian proteins, broadly inhibiting this pathway may result in significant adverse effects. Therefore, rather than focusing on individual SUMO pathway components, we examined the overall activation of the SUMO pathway across multiple tumor types. Remarkably, our investigation of 33 types of tumors revealed that nearly all tumors exhibited pronounced SUMO pathway activation. Furthermore, differential SUMO pathway activation has been observed across pathological subtypes within the same organ. These findings were corroborated by subsequent immunohistochemical analyses using tumor tissue microarrays. Collectively, these results suggest that SUMO pathway activation plays a role in tumor progression, and targeting this pathway could offer a promising approach for cancer treatment.

It is well established that oxygen levels influence protein SUMOylation, particularly in neurons, where it serves as a key neuroprotective mechanism following ischemia (Peters et al., 2017). In this study, we exposed four types of tumor organoids to hypoxic (5% O_2_), normoxia (21% O_2_), and hyperoxia (40% O_2_) conditions to investigate the effects of oxygen on SUMOylation. Our findings revealed that SUMO1 conjugate levels remained relatively stable across different oxygen concentrations. In contrast, the SUMO2/3 conjugates exhibited a significant increase under hypoxic conditions and a marked decrease under hyperoxic conditions. This observation is consistent with the understanding that SUMO1 conjugation is associated with delayed stress responses (Zhou et al., 2023), whereas SUMO2/3 conjugation occurs rapidly, often within 10 min, reflecting an acute stress response. These results provide further evidence supporting the role of SUMOylation in tumor organoids under various oxygen conditions.

Hypoxia is a critical mechanism that drives tumor resistance to chemotherapy (Chen et al., 2023). A key mediator of this resistance is Hypoxia-Inducible Factor-1α (HIF-1α), which is stabilized under low oxygen conditions. HIF-1α activation promotes the expression of genes involved in angiogenesis, invasion, metastasis, metabolic reprogramming, and cell survival, collectively contributing to therapy resistance. Our findings demonstrate that hypoxia decreases the sensitivity of tumor organoids to chemotherapeutic agents by upregulating SUMOylation, potentially intersecting with HIF-1α-mediated pathways. Conversely, exposure to hyperoxia enhanced chemosensitivity by downregulating SUMOylation levels. These results indicate that hyperoxia may serve as a straightforward and feasible approach to improving tumor chemosensitivity. However, directly translating the hyperoxia effects observed *in vitro* (40% O_2_) to clinically applicable and safe regimens require careful consideration, due to differences between *in vitro* pO_2_ levels and the physiological and pathophysiological pO_2_ ranges in tissues and tumors *in vivo*. Systematic *in vivo* studies are essential to fully evaluate the therapeutic potential and refine the application of oxygen modulation.

We subsequently examined the impact of disrupting the SUMOylation pathway in four different tumor organoid models. UBC9, the sole E2-conjugating enzyme in the SUMOylation pathway (Müller et al., 2001), was targeted to effectively suppress SUMOylation. Our results demonstrated that UBC9 inhibition significantly inhibited the growth of all four tumor organoid models. However, the proliferation of many stem cells and progenitor cells, which are crucial for tissue regeneration and repair, depends on UBC9-mediated SUMOylation (Kalamarz et al., 2012). To assess the potential side effects of UBC9 inhibition, we introduced fetal mouse-derived liver and kidney organoids. These findings revealed that UBC9 inhibition induced rapid growth failure, cell cycle arrest, and extensive apoptosis in these organoids and cells. These results suggest that UBC9 may not be a suitable target for tumor therapy because of its critical role in cellular proliferation and survival.

Bioinformatics analysis revealed that SENP5 is specifically expressed in certain tumors and is associated with overall survival in cancer patients, indicating its potential as a therapeutic target. Here, we observed that SENP5 overexpression significantly enhanced the growth of CAKI-2 cells and breast cancer organoids while exerting minimal effects on lung adenocarcinoma organoids, HepG2, and fetal mouse-derived liver and kidney organoids. These findings suggest that although SENP5 overexpression leads to different outcomes across various tumor types, it is unlikely to produce significant adverse effects on normal tissues.

Although SENP5 overexpression has little effect on normal progenitor cells, it promotes the proliferation of certain tumors, which conflicts with the goal of cancer therapy. Therefore, the challenge lies in harnessing the therapeutic potential of targeting SENP5, while minimizing its adverse effects. Further bioinformatic analysis revealed that SENP5 specifically activates the cell cycle, which may explain its role in driving tumor proliferation. Theoretically, hypoxia can induce temporary dormancy in tumor cells, contributing to chemotherapy resistance (Kumar et al., 2022; Barrios et al., 2022; Pinzón-Daza et al., 2017; Lv et al., 2015). We hypothesized that SENP5 overexpression might enhance the sensitivity of tumor cells to chemotherapeutic agents, a hypothesis that was validated by subsequent *in vitro* experiments. An intriguing question arising from our findings is whether tumors originating from tissues with typically higher baseline oxygen demand (e.g., brain, heart, kidney) might exhibit greater sensitivity to SENP5 overexpression followed by chemotherapy, compared to tumors from tissues with lower intrinsic demand. While our current organoid models included renal cancer, a systematic comparison across tumors derived from tissues with varying oxygen consumption rates represents an important avenue for future investigation to refine potential SENP5-based therapeutic strategies.

Our analysis of the relationship between the key members of the SUMO pathway and functional pathways suggests that SENP5 may specifically inhibit the RAS/MAPK pathway. Previous studies have shown that SUMOylation, particularly involving SUMO2/3, primarily affects the localization of target proteins, formation of protein complexes, and phosphorylation of adjacent amino acid residues, rather than altering overall protein expression levels. We hypothesized that SENP5 exerts its inhibitory effect on the RAS/MAPK pathway by modulating the phosphorylation of specific residues. This hypothesis is grounded in the established role of SUMO2/3 conjugation in primarily modulating protein-protein interactions, subcellular localization, the assembly of functional complexes, and the phosphorylation status of nearby residues, rather than directly altering the total abundance of target proteins (Nan et al., 2022; Bragado et al., 2022). To test this hypothesis, we assessed the impact of SENP5 overexpression on the phosphorylation levels of four key RAS/MAPK pathway members: Raf1, MEK, ERK, and ETS1. Our results showed that SENP5 decreased the phosphorylation levels of MEK, ERK, and ETS1 while increasing the phosphorylation of Raf1. Notably, MEK and ETS1 have been previously identified as SUMOylation targets, whereas Raf1 and ERK have not. Thus, we speculate that SENP5 may reduce the phosphorylation levels of MEK and ETS1 via deSUMOylation, leading to overall inhibition of the RAS/MAPK pathway. However, the mechanism by which SENP5 increases Raf1 phosphorylation remains unclear. To further validate the specificity of SENP5’s inhibitory effect on the RAS/MAPK pathway, we examined the phosphorylation levels of AKT, a known SUMOylation target in the EGFR downstream PI3K/AKT pathway. Our findings showed that SENP5 did not alter AKT phosphorylation, suggesting that the inhibitory effect of SENP5 on the RAS/MAPK pathway is specific.

Our analysis of drug sensitivity using the Genomics of Drug Sensitivity in Cancer (GDSC) (Geeleher et al., 2014) database revealed a potential correlation between SENP5 expression and sensitivity to 17-AAG, RDEA119, Trametinib, and Selumetinib, suggesting that these could be potential therapeutic agents targeting SENP5. Our experimental results confirmed that the overexpression of SENP5 significantly increased the sensitivity of tumor organoids to these four drugs *in vitro*.

In summary, our study revealed that the activation of the SUMOylation pathway is a prevalent event in tumor progression, but indiscriminate interference with this pathway can lead to severe side effects in normal tissues. For the first time, we demonstrated that SENP5 overexpression can promote cell cycle progression in organoid models derived from four different tumors, thereby restoring sensitivity to chemotherapy in previously resistant tumor organoids. Importantly, SENP5 overexpression did not cause significant adverse effects in normal tissues. The tumor-specific action and minimal side effects associated with SENP5 make it a promising target for cancer therapy. However, the extrapolation of our *in vitro* organoid findings, particularly regarding oxygen effects and SENP5-mediated sensitization, to the complex *in vivo* tumor microenvironment necessitates further validation in appropriate animal models.

## Data Availability

The datasets presented in this study can be found in online repositories. The names of the repository/repositories and accession number(s) can be found in the article.
