# Supplementary figures and images for: SUMOylation involved in malignant progression of multiple tumors and SENP5 may improve the chemotherapy sensitivity of hypoxic tumors
Source: Front Pharmacol. 2025 Nov 5;16:1648271. doi: 10.3389/fphar.2025.1648271 (PMC12626937; doi:10.3389/fphar.2025.1648271)

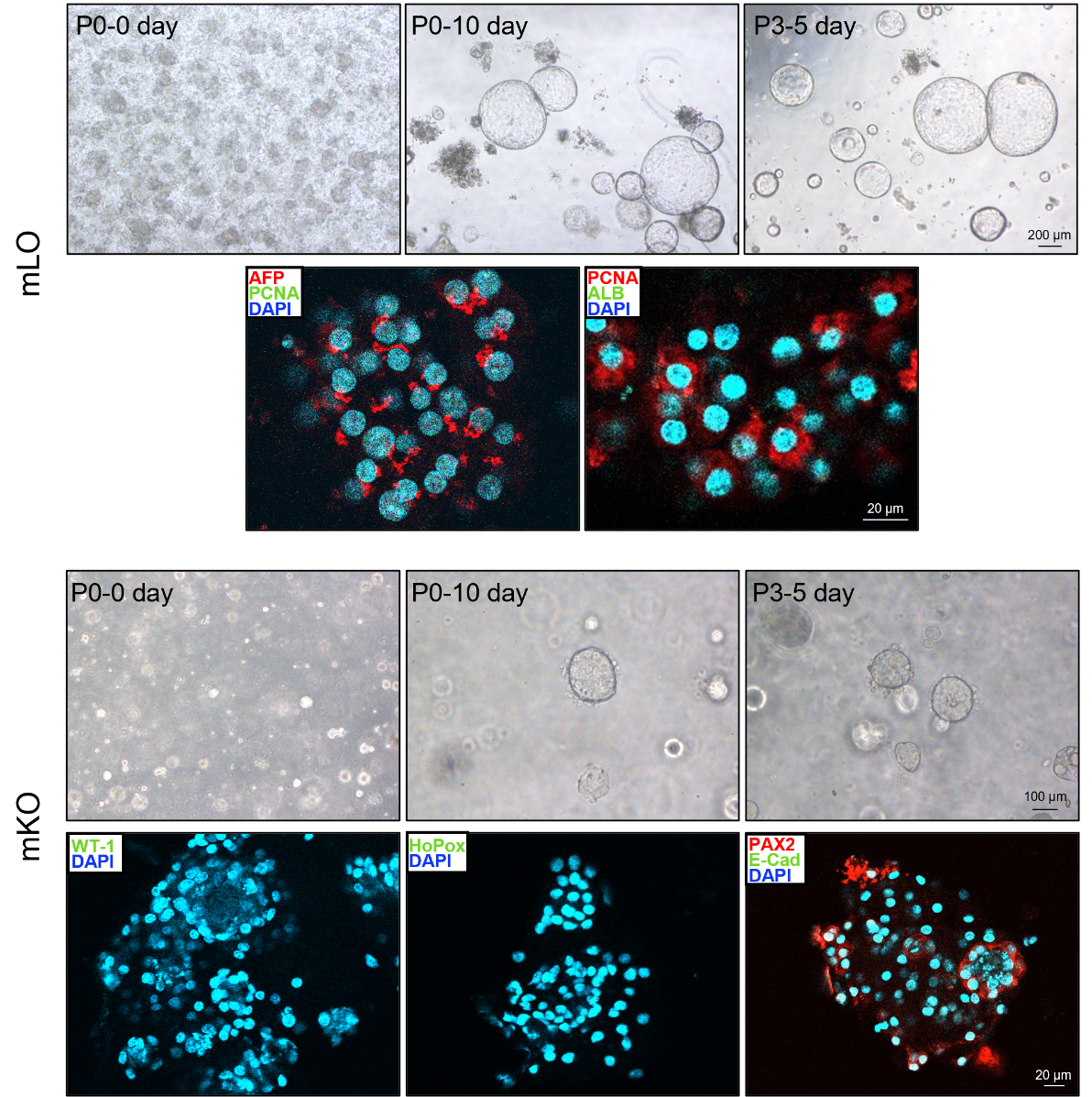

Supplement: Supplementary file 2 [file Image1.tif]
